# Dynamics of recovery sleep from chronic sleep restriction

**DOI:** 10.1093/sleepadvances/zpac044

**Published:** 2022-11-30

**Authors:** Jacob R Guzzetti, Siobhan Banks

**Affiliations:** Behaviour-Brain-Body Research Centre, University of South Australia, Adelaide, South Australia, Australia; Behaviour-Brain-Body Research Centre, University of South Australia, Adelaide, South Australia, Australia

**Keywords:** chronic sleep restriction, recovery sleep, cognitive performance, mood, sleepiness, Psychomotor Vigilance Task

## Abstract

Sleep loss is common in our 24/7 society with many people routinely sleeping less than they need. Sleep debt is a term describing the difference between the amount of sleep needed, and the amount of sleep obtained. Sleep debt can accumulate over time, resulting in poor cognitive performance, increased sleepiness, poor mood, and a higher risk for accidents. Over the last 30 years, the sleep field has increasingly focused attention on recovery sleep and the ways we can recover from a sleep debt faster and more effectively. While there are still many unanswered questions and debates about the nature of recovery sleep, such as the exact components of sleep important for recovery of function, the amount of sleep needed to recover and the impacts of prior sleep history on recovery, recent research has revealed several important attributes about recovery sleep: (1) the dynamics of the recovery process is impacted by the type of sleep loss (acute versus chronic), (2) mood, sleepiness, and other aspects of cognitive performance recover at different rates, and (3) the recovery process is complex and dependent on the length of recovery sleep and the number of recovery opportunities available. This review will summarize the current state of the literature on recovery sleep, from specific studies of recovery sleep dynamics to napping, “banking” sleep and shiftwork, and will suggest the next steps for research in this field.

This paper is part of the David F. Dinges Festschrift Collection. This collection is sponsored by Pulsar Informatics and the Department of Psychiatry in the Perelman School of Medicine at the University of Pennsylvania.

**Statement of Significance**

Many people in today’s 24/7 society do not get sufficient sleep. This may be due to a sleep disorder, work schedules, career commitments, or any one of the many other reasons. Without adequate recovery a sleep debt can accumulate, increasing the risk for accidents and poor health. Professor David Dinges’ seminal work investigating recovery sleep has greatly influenced our understanding of sleep–wake dynamics and recovery sleep. Dinges’ work has inspired a generation of research on sleep debt and recovery dynamics and led to workplace policy change across many industries ensuring more productive and safe work schedules. This review highlights Dinges’ research with a particular focus on chronic sleep restriction and recovery sleep dynamics.

## Introduction

The prevalence of chronic sleep restriction in our 24/7 society is increasing with adults sleeping an average <7 h most nights [1, 2]. Chronic sleep restriction can be defined as obtaining less sleep than an individual needs for multiple nights in a row. This is distinct from total sleep deprivation which describes no sleep at all for an extended period of time [3]. As well as sleep duration, sleep behaviors are also changing, with frequent cycling between short sleep on workdays and extended sleeps on days off and weekends as individuals try to recover [4]. Experiments in healthy humans have demonstrated that chronic sleep restriction results in cognitive performance deficits and increased sleepiness that accumulates over days [5–8]. These deficits can accumulate to similar levels found for several days of total sleep deprivation [8]. While many studies have confirmed that neurobehavioural and broader human physiological systems impairment accrues with sleep loss, the dynamics of the recovery process are only beginning to be understood.

It was nearly 60 years ago that Kleitman [9] suggested that “sleep debts” are “liquidated” by extending recovery sleep duration (p. 317) however, research is yet to address gaps in knowledge pertaining to the understanding of recovery sleep and its dynamics. For example, it is not yet known what the rates of homeostatic sleep drive buildup with sleep restriction and dissipation with recovery sleep are. Further, research to date has not demonstrated which components of sleep architecture are important for recovery of function nor developed recommendations regarding behavioral changes that might ensure an adequate number of days off duty for recovery from work schedules. This is likely in part due to the ways recovery has been studied and the complexity of the recovery process after chronic sleep restriction. Initially, recovery was studied only after total sleep deprivation, but the recovery dynamics are quite different after chronic sleep restriction [5]. To study recovery, participants must first undergo a period of chronic sleep restriction which requires lengthy, complex laboratory studies. As a result, few of these study types are undertaken. Understanding recovery dynamics is important, not only for individuals who need to resolve a sleep debt, but also to ensure organizations create work schedules that allow for adequate recovery time.

David Dinges seminal work investigating recovery sleep has greatly influenced the understanding of sleep–wake dynamics and recovery sleep. This review will feature and highlight his research and focus on human studies of recovery sleep dynamics. The review will summarize both seminal and recent literature on human studies of sleep restriction and recovery and discuss the recovery value of short sleeps, such as naps, split sleep opportunities, and banking sleep.

### Impact of sleep debt on cognitive performance

Chronic sleep loss without adequate recovery sleep leads to what is referred to as “sleep debt” [10, 11]. Sleep debt is common in many segments of society including new parents [12], shift workers [13], long-haul truck drivers [14], nurses [15], commercial pilots [16], and astronauts [17]. Chronic sleep loss is associated with behavioral risks that include increased errors, traffic accidents, injuries, poor team performance, and burnout [18, 19].

Webb and Agnew [10] suggested that a “sleep debt” underpinned their finding of increased sleep duration following the restriction of sleep to either 2 or 4 h a night. They suggested an increased sleep duration may occur in response to a sleep debt, alluding to the corrective process now known as recovery sleep. These results built on the findings of Dement et al. [20] who found that supplemental REM sleep was critical to the reversal or recovery of the effects of restricted REM sleep.

Dinges et al. [7] conducted the first study of its kind to examine the impact of chronic sleep restriction on cognitive performance, sleepiness, and mood. Under controlled laboratory conditions, participants were subjected to restricted sleep (5 h per night) over 7 days. Results demonstrated that deficits in cognitive performance, sleepiness, and mood accumulated over days of chronic sleep restriction and that these deficits failed to be corrected after one recovery sleep opportunity of 10 h. This study highlights the complex relationship between sleep debt and recovery sleep. Continuing this work, Dinges et al. [8] conducted another chronic sleep restriction laboratory study comparing the cognitive performance consequences of either 4, 6, or 8 h’ sleep per day for 14 days. This study would become a seminal work revealing that even relatively moderate amounts of sleep restriction for a short period could result in cognitive performance deficits similar to two nights without any sleep. This study also found that subjective sleepiness ratings largely stabilized after 2–3 days, despite continued cognitive performance decline [8]. These observations suggest that the sleep debt, which affects a large portion of the population, comes at a neurobehavioral cost that may be difficult to detect.

Further, Belenky et al. [5] conducted a similar study examining cognitive performance under different sleep doses (3, 5, 7, or 9 h) for 7 days and 3 subsequent days of 8 h’ recovery sleep. While cognitive performance declined in the 3-h group, it appeared to plateau in the 7-h group. Evaluation of cognitive performance during the recovery period yielded interesting findings: following the first recovery night, the 3-h group demonstrated an improvement in cognitive performance but only to a level consistent with that of the 5-h and 7-h groups. There was sustained cognitive impairment relative to baseline for each of the 3-h, 5-h, and 7-h groups implying that 3 days of 8 h’ sleep was insufficient for complete recovery [5].

### Weekend recovery sleep and repeated periods of restriction and recovery

The 5 days for work and 2 days for rest structure of the week is a socially engrained work/rest interval [21]. This work configuration has resulted in widespread reports of shorter, restricted weekday sleep with longer, extended sleep on at least one weekend night (or day off from work), representing a cyclic schedule of chronic sleep restriction and recovery [4]. While this is a common sleep pattern for millions of people [4] it has rarely been studied.

**Figure 1.**
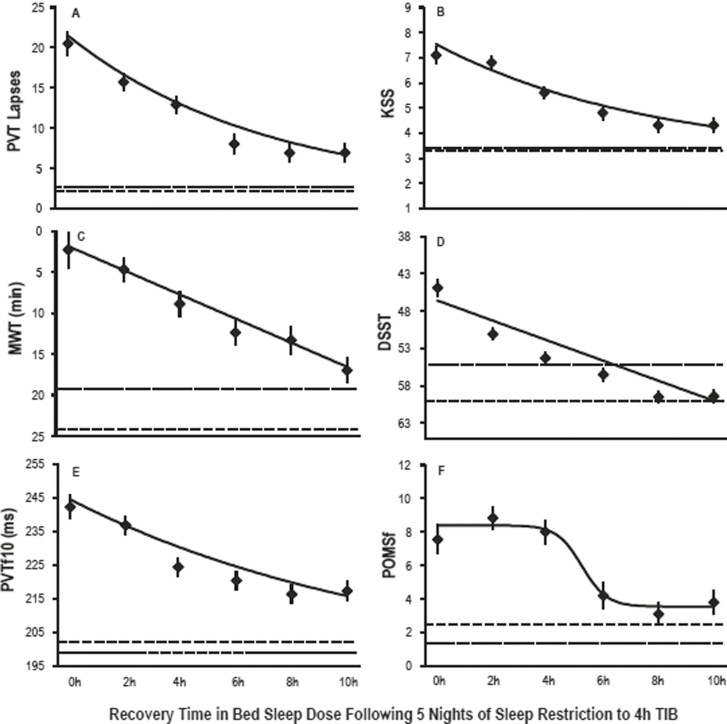
Recovery of neurobehavioral outcomes as a function of increasing time in bed for sleep. Least squares means (± SEM) are shown as diamonds for each recovery sleep dose. The horizontal dotted lines show baseline night sleep data. The horizontal dashed lines show the control group means on day 8 where they had 10 h time in bed for sleep. All neurobehavioral outcomes showed improvement as recovery sleep doses increased. These data show that greater time in bed for sleep resulted in greater improvements in neurobehavioral performance. Figure reproduced from Banks S, Van Dongen HP, Maislin G, Dinges DF. Neurobehavioral dynamics following chronic sleep restriction: dose-response effects of one night for recovery. Sleep. 2010;33(8):1013–1026, by permission of Oxford University Press.

**Figure 2.**
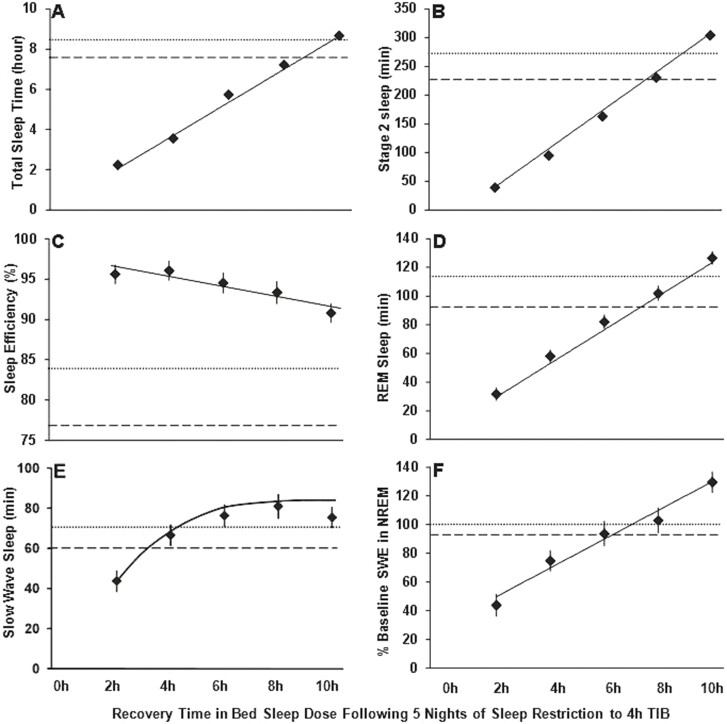
Recovery of sleep variables as a function of increasing time in bed dose. Least squares means (± SEM) are shown as diamonds for each recovery sleep dose group. The horizontal dotted lines show baseline night sleep data. The horizontal dashed lines show the control group means on day 8 where they had 10 h time in bed for sleep. Increasing recovery time in bed sleep dose increased total sleep time (TST), stage 2 sleep, rapid eye movement sleep (REM), slow wave sleep (SWS), and amount of slow wave energy (SWE) or delta power in the EEG. Sleep efficiency decreased with increasing time in bed. These data indicate that the amount of sleep time and delta power in the non-REM EEG are important for recovery of function. Figure reproduced from Banks S, Van Dongen HP, Maislin G, Dinges DF. Neurobehavioral dynamics following chronic sleep restriction: dose-response effects of one night for recovery. Sleep. 2010;33(8):1013–1026, by permission of Oxford University Press.

One of the few studies to systematically examine the amount of sleep needed to recover a typical work week of chronic sleep restriction was by Banks et al. [22]. They sought to investigate the magnitude of recovery that could be achieved in a single night after five nights of sleep restriction. Neurobehavioral performance was assessed during 5 days of sleep restriction to 4 h’ time in bed a night, and then after a recovery night of either 0, 2, 4, 6, 8, or 10 h’ time in bed for sleep. A control group with a 10-h sleep opportunity each night of the study was also examined. Analyses assessed recovery to baseline for all groups including control. It was found that ability to maintain wakefulness and cognitive throughput improved as sleep duration, sleep stages, and sleep intensity increased across the recovery sleep doses (see Figure 1). However, vigilant attention, subjective sleepiness, and subjective mood did not follow this same pattern. Participants did not fully recover compared to their baseline or the control group even with 10 h’ time in bed for sleep. This work suggests that different metrics may recover at different rates, with different recovery trajectories. The lack of full recovery has implications for individuals if they are re-exposed to further periods of sleep restriction as is typical with the cyclic weekday/weekend pattern. These results also suggest that the more time available for sleep after sleep restriction, the greater the recovery of neurobehavioural function. The 10 h’ time in bed recovery condition had significantly more total sleep time (sleep duration), stage 2 sleep, and percentage of slow wave energy (or sleep intensity) on the recovery night than at baseline night (see Figure 2). It has been previously suggested that sleep intensity and sleep duration are only “marginally related, and that “sleep loss is primarily recovered by increasing sleep intensity and not necessarily by sleep duration” [23]. The data from Banks et al. [21] does not support this and would suggest that both sleep intensity and sleep duration are important for recovery of neurobehavioural function following chronic sleep restriction.

The role of preexisting sleep debt on the subsequent response to sleep restriction was addressed in a preliminary study by Banks et al. [24]. This study investigated whether a single night of sleep restriction to 4 h’ time in bed following partial recovery from a sleep debt resulted in the same degree of neurobehavioral deficit as that found after a single night of sleep restriction to 4 h’ time in bed following a period without sleep debt. Healthy individuals participated in a laboratory-controlled protocol, where they underwent two nights of baseline sleep of 10 h’ time in bed; Followed by five nights of sleep restriction to 4 h’ time in bed a night; Then a recovery night of between 8 and 12 h’ time in bed; Followed by another night of sleep restriction to 4 h’ time in bed. Change scores were calculated between the second baseline night and first night of sleep restriction (assessment 1; acute sleep restriction after no sleep debt), and between the recovery night and subsequent night of sleep restriction (assessment 2; acute sleep restriction after sleep debt). Vigilant attention at assessment 2, acute sleep restriction after sleep debt, was nearly twice the impairment compared to that at assessment 1, acute sleep restriction after no sleep debt. Thus, when recovery from sleep debt is incomplete, neurobehavioral vulnerability to further sleep restriction appears to be disproportionately increased.

This pattern of weekday sleep restriction and weekend sleep extension has also been examined in the context of metabolic [25] and immune function [26] as an array of negative health outcomes are known to result from chronic sleep restriction [27]. Depner et al. [28] examined if ad libitum weekend recovery sleep would prevent metabolic dysregulation when re-exposed to chronic sleep restriction. During the weekend, participants slept approximately 1 h more than baseline, but during chronic sleep restriction following the weekend, the circadian phase was delayed, and after-dinner energy intake and body weight increased. Overall, they found that weekend recovery sleep did not protect against metabolic disruption during sleep restriction the subsequent week. There were residual effects of the first period of sleep restriction on the second period, regardless of the intervening weekend recovery sleep.

Simpson et al. [26] examined this same model of cyclic weekday restriction and weekend recovery sleep on stress and immune function. They examined the impact of three periods of sleep restriction to 4 h’ time in bed a night for 5 days (weekdays) with 2 days recovery of 8 h per night (weekend) on physiological markers of stress. Results showed that physiological stress responses remained activated with repeated exposures to sleep restriction and “weekend” recovery. Immune function was increased during sleep restriction and remained increased after recovery sleep in weeks one and two. These results provide evidence that patterns of sleep restriction and recovery have implications for immune function and given the awareness that chronic low-grade inflammation can increase risk for cardiovascular and metabolic disease [29] these patterns of insufficient sleep may pose a significant health risk.

Collectively, evidence from the above studies suggest that even after extended periods of recovery sleep, recent exposure to chronic sleep loss can make an individual more vulnerable to the effects of re-exposure to sleep restriction. Weekends and time off appear to not provide much protection when cycling between periods of short sleep and longer recovery sleep. Despite intermittent opportunities for recovery sleep, individuals exposed to work schedules that regularly restrict sleep may become increasingly vulnerable to the adverse effects of sleep loss on performance. Therefore, prior sleep-wake history may greatly impact an individual’s response to future sleep loss.

### “Banking” sleep and extending sleep to maximize recovery

Banking sleep is characterized by extending habitual sleep duration in advance of a period of sleep restriction [30]. In a seminal study, Rupp et al. [30] sought to examine the impact of extended habitual sleep duration in advance of a period of chronic sleep restriction on performance. Participants had a week of either habitual (7 h a night) or extended (10 h a night) sleep opportunities before undergoing sleep restriction of 3 h a night for 7 days. This was followed by 5 days of 8 h’ time in bed a night for recovery sleep. Participants in the extended sleep condition before the chronic sleep restriction showed less performance impairment compared to those in the habitual sleep condition. The additional sleep in the extended condition improved the participant’s resiliency to the sleep restriction. Further, performance deficits were more quickly resolved in extended sleep group during the recovery phase, suggesting that both performance impairment under sleep restriction and the time course of subsequent recovery are influenced by prior sleep.

These findings demonstrate that there is a long-term effect of prior sleep history that can increase resilience or vulnerability to sleep restriction. Indeed, Banks et al. [24] found that when recovery from sleep debt is incomplete, cognitive performance during subsequent sleep restriction appears to be disproportionately increased (i.e. increased vulnerability).

### Recovery for workers around the clock

Shiftworkers, particularly nightshift personnel, are often faced with the distinct challenges of daytime sleep and circadian misalignment. Sleep during the day is difficult due to the circadian system’s drive for wakefulness. Sleep during the day is often shorter and of reduced quality [31]. Therefore, shiftworkers face increased vulnerability to sleep debt accrual, and a suboptimized opportunity to recover the debt. Additionally, the opportunity for recovery is often restricted as a byproduct of successive long shift (12 h+) duration in shiftworkers in operational environments. In a field study of nurses working three sequential 12-h night shifts, Geiger-Brown et al. [32] found an average sleep duration of 5.4 h between shifts, which was extended by only 0.67 h, on average, after the third shift, demonstrating the vulnerability to sleep debt and barriers to recovery in nightshift personnel. This is problematic as Jay et al. [33] reported that neurobehavioral functions are not properly recovered when recovery sleep durations are restricted.

In a novel, a split-sleep dose-response study involving a range of scenarios with chronically reduced nocturnal sleep, augmented with a diurnal nap, Mollicone and colleagues [34] showed that cognitive performance declined at the same rate regardless of whether sleep was consolidated or split into a nocturnal anchor sleep and nap. Cognitive performance was primarily a function of total time in bed per 24 h, with less total time in bed consistently resulting in a greater accumulation of performance impairment and subjective sleepiness across days. Provided total sleep time is the same, sleep can be split into two periods or consolidated in one. These findings have implications for individuals with work schedules that rarely permit long nocturnal sleep episodes. For these individuals, results suggest that splitting the sleep can provide adequate recovery to maintain performance.

Supplementing sleep in the anticipation of sleep loss is a common practice by many shiftworkers. Geiger-Brown et al. [32] found that nearly 75% of nightshift nurses reported napping prior to their first night shift. Although prophylactic napping, a concept originally proposed by Orne and Dinges [35], was for some time not considered to be possible “because sleep could not be stored^(p131^ [36]^)^,” considerable real-world evidence supports the usefulness of the approach as does the experimental work on banking sleep described above by Rupp and colleagues [30]. Seminal work by Dinges et al. [35, 37] investigated the recovery benefit of prophylactic naps in sleep-deprived adults. The research included five different 2-h nap opportunities during 2.5 days without sleep. One of these opportunities involved a nap on the first afternoon, after only 6 h of wakefulness (i.e. before 46 h of sustained wakefulness). Subjects who had undertaken a prophylactic nap on the first afternoon demonstrated improved reaction time on the psychomotor vigilance task, however it was not evident until 10 h post the nap (i.e. during the first full day of sleep loss). Once sleep deprivation was present, the positive effects of the nap on performance were evident within an hour after the nap and were sustained for between 6 and 30 h post nap [35]. Dinges concluded that afternoon naps, including those taken prophylactically before sustained wakefulness, have beneficial effects on performance and sleepiness up to 12 h after the naps.

## Conclusions and Summary

In summary, as sleep loss is common and many individuals do not get adequate sleep, it is important to understand the dynamics of the recovery process. It is clear from the literature reviewed here that recovery from chronic sleep restriction is a complex process that is not possible with one or two nights of extended sleep. The pattern of weekend catch-up sleep does not permit full recovery of lost sleep or neurobehavioural function and does not provide protection if re-exposed to chronic sleep restriction. When recovery from chronic sleep restriction is incomplete, performance during subsequent sleep restriction appears to be disproportionately increased (i.e. increased vulnerability to the impact of sleep restriction). This indicates that prior sleep history is an important factor in how an individual will respond to sleep restriction. It is also clear from the reviewed literature that both sleep duration and sleep intensity is important for recovery. It is not evident that one sleep stage or component of sleep is more important or vital for recovery than another. This is echoed in studies that split sleep where the total amount of sleep obtained over a 24-h period is the important factor to maintain performance. Indeed, naps and short sleeps can supplement recovery when extended consolidated sleeps are not possible. Recovery is important to reverse the negative effects of sleep restriction and to maintain neurobehavioral function and health. Effectively managing recovery to ensure people do not develop a significant sleep debt, particularly those workers who have limited opportunities for recovery sleep, could have major impacts for wellbeing, increasing productivity and help to reduce road crashes and workplace accidents. Relatedly, studies examining the impact of chronic sleep restriction and associated recovery sleep dynamics in the sleep disorders patient population are in critical demand.
